# Monitoring Residual Disease in the Ph-Negative Myeloproliferative Neoplasms Post-Allogeneic Stem Cell Transplantation: More Mutations and More Methodologies

**DOI:** 10.3389/fonc.2014.00212

**Published:** 2014-08-08

**Authors:** Stephen E. Langabeer, Karl Haslam, Eibhlin Conneally

**Affiliations:** ^1^Cancer Molecular Diagnostics, St. James’s Hospital, Dublin, Ireland; ^2^Department of Haematology, St. James’s Hospital, Dublin, Ireland

**Keywords:** myeloproliferative neoplasms, allogeneic stem cell transplantation, residual disease, *JAK2* V617F, *CALR*, next generation sequencing, digital PCR

The Philadelphia chromosome-negative myeloproliferative neoplasms (MPN) are a group of clonal hematopoietic diseases characterized by bone marrow proliferation of one or more of the myeloid cell lineages with no marked alterations in cellular maturation. MPN classically comprise the clinically and pathologically related polycythemia vera (PV), essential thromobocythemia (ET), and primary myelofibrosis (PMF). Identification of the *JAK2* V617F mutation has revolutionized the molecular diagnosis of MPN as this mutation is present in >95% of patients with PV and in 50% of patients with ET and PMF. In PV and ET, the potential exists for the disease to transform to a myelofibrotic stage and, together with PMF, transformation to acute myeloid leukemia (AML). The introduction of small molecule inhibitors that abrogate both normal and mutant JAK protein intracellular signaling in PMF has undoubtedly been a major advance in the treatment of these types of malignancies (1). Nevertheless, the only potentially curative option for these diseases, particularly PMF, is allogeneic stem cell transplantation (ASCT). Though ASCT was previously considered only for those patients with advanced or transformed disease, improvements in candidate patient selection and stratification, timing of transplantation, and conditioning regimens have significantly reduced the transplant related morbidity and increased the overall survival for MPN patients undergoing this procedure (2). However, as relapse is a major cause of treatment failure post-ASCT with salvage options limited and subsequent outcome relatively poor, identification of those patients at high-risk of relapse would be highly desirable, potentially enabling therapeutic intervention before overt relapse.

Conventionally, donor chimerism status is used to assess engraftment post-ASCT for hematological malignancies. Comparison of donor and recipient profiles can be achieved by either short tandem repeat (STR) analysis or quantitative polymerase chain reaction (qPCR) possessing sensitivities of 1–2%, with post-ASCT surveillance performed at one to three-monthly intervals. Post-ASCT monitoring utilizing additional patient-specific markers is most likely to provide a more beneficial, personalized profile with this approach already applied to many MPN patients undergoing ASCT. Early studies demonstrated the achievement of a molecular remission in *JAK2* V617F-positive MPN post-ASCT using qualitative PCR (3). Development of more sensitive, *JAK2* V617F-specific qPCR methodologies capable of detecting one mutant allele in 10^4^ wild type copies has subsequently been shown to provide information on the rate of disease eradication and the identification of patients, at defined time points post-ASCT, at an increased risk of relapse (4–6). These sensitive *JAK2* V617F qPCR assays have also been shown to be of value in triggering adoptive immunotherapies such as donor lymphocyte infusions that are able to elicit a graft-versus-tumor effect both preemptively and for salvage, post-relapse (7, 8). Even so, such qPCR methodologies require optimization across platforms and rigorous attention to reliability and sensitivity to ensure continued clinical utility (9). Mutations of *MPL* that encodes the receptor for thrombopoietin are also recurrent in ET and PMF but at a much lower frequency than the *JAK2* V617F. In those reported *MPL*-mutation positive MPN who have undergone ASCT, rapid clearance of the *MPL* W515L mutation correlated well with peripheral blood counts and donor chimerism status (10).

More recently, whole exome sequencing has identified insertion and/or deletion mutation in *CALR*, a gene that encodes the endoplasmic reticulum-associated, calcium binding protein calreticulin. These mutations, which occur exclusively in *CALR* exon nine, appear not to be found in PV, and are present in up to 80% of ET and PMF patients who are *JAK2* V617F-and *MPL*-negative (11, 12). As *CALR* mutations are likely initiating events in MPN pathogenesis, the possibility arises to assess these mutations as markers of residual disease in MPN patients post-ASCT. An initial assessment of *CALR* mutant allele burden, using semi-quantitative PCR fragment analysis, has shown that eradication and persistence of *CALR* mutations mirror donor chimerism status providing initial validation for future prospective serial monitoring of *CALR* mutations post-ASCT (13).

Apart from the common *JAK2* V617F, *MPL*, and *CALR* mutations, several other recurrent mutations are observed in MPN but are also present in the myelodysplastic syndromes (MDS), MDS/MPN syndromes, and AML limiting their diagnostic utility but affording the potential for their use as markers of residual disease. These include mutations of *TET2, ASXLI, CBL, NRAS*, and *EZH2*. Rarely, mutations within some of these genes have been demonstrated in normal individuals (14) advocating caution when employed as markers of residual disease. Next generation sequencing (NGS) technologies now afford the opportunity to identify pathogenic mutations in multiple amplicons from these and many other genes simultaneously. A recent proof of principle study has utilized NGS to identify molecular mutations in MDS/MPN patients pre-ASCT with detection of the corresponding mutation in the post-ASCT period predictive of relapse (15). Whereas qPCR techniques provide relative quantitation of target amplicons, the emerging technology of digital PCR (dPCR) allows the absolute quantitation of mutant alleles with very small fold changes quantifiable. This approach has been applied to detect and monitor extremely low levels of *BCR-ABL1* transcripts in chronic myeloid leukemia and acute lymphoblastic leukemia patients who have achieved deep molecular responses with tyrosine kinase inhibitor therapy (16, 17).

Whether NGS technologies will be of value in routine practice in the post-ASCT setting remains to be proven. Currently, limits of detection require improvement in order to approach those achieved by qPCR necessary for clinical application (Figure 1). Furthermore, issues such as intra- and inter-laboratory will require addressing for both NGS and dPCR; preliminary studies with NGS have demonstrated technical feasibility and concordance in the diagnostic setting (18). These emerging methodologies might soon be applied to identify and monitor mutational events enabling a distinct, personalized profile in those MPN patients undergoing ASCT. The goal would be the prompt recognition of those patients with an increased risk of relapse in which early therapeutic intervention is warranted, ultimately leading to gains in long-term disease-free and overall survival.

**Figure 1:**
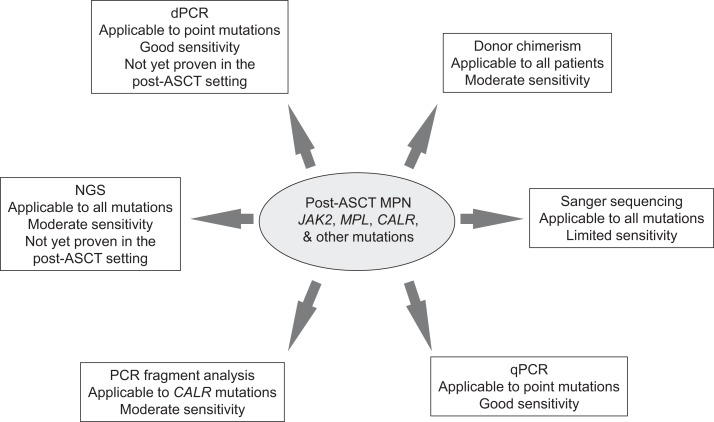
**Summary of methodologies for monitoring MPN-specific mutations post-allogeneic stem cell transplantation**. ASCT, allogeneic stem cell transplantation; MPN, myeloproliferative neoplasm; qPCR, quantitative polymerase chain reaction; NGS, next generation sequencing; and dPCR, digital polymerase chain reaction.

## Conflict of Interest Statement

The authors declare that the research was conducted in the absence of any commercial or financial relationships that could be construed as a potential conflict of interest.
